# Assessment of The Dose-Response Relationship of Radiation-Induced Bystander Effect in Two Cell Lines Exposed to High Doses of Ionizing Radiation (6 and 8 Gy) 

**Published:** 2017-08-19

**Authors:** Mohammad Taghi Bahreyni Toossi, Sara Khademi, Hosein Azimian, Shokoufeh Mohebbi, Shokouhozaman Soleymanifard

**Affiliations:** 1 Medical Physics Research Center, Mashhad University of Medical Sciences, Mashhad, Iran; 2 Department of Medical Physics, School of Medicine, Mashhad University of Medical Sciences, Mashhad, Iran; 3 Department of Medical Physics and Biomedical Engineering, Faculty of Medicine, Tehran University of Medical Sciences, Tehran, Iran; 4 Department of Medical Physics, Omid Hospital, Mashhad, Iran

**Keywords:** Dose-Response Relationship, MRC5 Cell Line, Radiotherapy

## Abstract

**Objective:**

The dose-response relationship of radiation-induced bystander effect (RIBE) is controversial at high dose levels. The aim of the present study is to assess RIBE at high dose levels by examination of different endpoints.

**Materials and Methods:**

This experimental study used the medium transfer technique to induce RIBE. The cells were divided into two main groups: QU-DB cells which received medium from autologous irradiated cells and MRC5 cells which received medium from irradiated QU-DB cells. Colony, MTT, and micronucleus assays were performed to quantify bystander responses. The medium was diluted and transferred to bystander cells to investigate whether medium dilution could revive the RIBE response that disappeared at a high dose.

**Results:**

The RIBE level in QU-DB bystander cells increased in the dose range of 0.5 to 4 Gy, but decreased at 6 and 8 Gy. The Micronucleated cells per 1000 binucleated cells (MNBN) frequency of QU-DB bystander cells which received the most diluted medium from 6 and 8 Gy QU-DB irradiated cells reached the maximum level compared to the MNBN frequency of the cells that received complete medium (P<0.0001). MNBN frequency of MRC5 cells which received the most diluted medium from 4 Gy QU-DB irradiated cells reached the maximum level compared to MNBN frequency of cells that received complete medium (P<0.0001).

**Conclusion:**

Our results showed that RIBE levels decreased at doses above 4 Gy; however, RIBE increased when diluted conditioned medium was transferred to bystander cells. This finding confirmed that a negative feedback mechanism was responsible for the decrease in RIBE response at high doses. Decrease of RIBE at high doses might be used to predict that in radiosurgery, brachytherapy and grid therapy, in which high dose per fraction is applied, normal tissue damage owing to RIBE may decrease.

## Introduction

The term radiation-induced bystander effect (RIBE) refers to the occurrence of radiation effects in non-irradiated cells. RIBE has been extensively investigated in low-dose radiation and is one of the main interests in high dose radiation. It has been shown that RIBE is dependent on several parameters that include radiation dose (1), dose rate (2), dose fractionation (3,4), radiation quality (5), cell/tissue type and the investigated biological endpoints (6,10). However, it is not yet well understood how each of these parameters affect RIBE. Some studies report a direct relationship between RIBE level and dose, whereas others have reported an initial linear relation followed by a second phase horizontal line, which demonstrated RIBE saturation (11,12). A number of studies have indicated that the magnitude of damage induced in bystander cells (RIBE level) is independent of dose (9,11). 

Radiotherapy techniques such as brachytherapy, stereotactic radiosurgery, intraoperative radiotherapy, and grid therapy apply high doses of radiation. Therefore, it is important to study RIBE at these doses. Soleymanifard et al. (4,13) have reported that RIBE in QU-DB cells increased when radiation dose rose from 0.5 to 4 Gy. However, they observed a decrease in RIBE when the radiation dose increased to higher levels. They also observed that RIBE increased in MRC5 cells which received conditioned medium extracted from 0.5 and 2 Gy QU-DB irradiated cells. When the radiation dose reached 4 Gy, RIBE decreased. These results indicated RIBE dose-response relationship at high and low doses were unexpectedly different. We designed the present study to investigate whether the results obtained by Soleymanifard et al. (13) were reproducible. Therefore, we intended to examine other end-points according to the colony, MTT, and micronucleus assays. 

There are three hypotheses to explain the reduction of RIBE at high doses. According to Gow et al. (2), the intensity of bystander signals produced by target cells increases as the dose increases; however, at high dose which bystander signals rise, a negative feedback acts to reduce RIBE. The second hypothesis according to Mackonis et al. (14), states that high amounts of bystander signals generated by target cells at high doses activate a repair mechanism in viable cells which decreases RIBE. The third hypothesis according to Blyth and Sykes (9) states that the reduction of bystander signals produced by target cells, due to target cells’ death at a high dose, causes RIBE decrease. We designed this study to examine the three hypotheses which might explain the reduction in RIBE at 6 and 8 Gy for QU-DB cells and 4 Gy for MRC5 cells. For this purpose, we added fresh medium to the conditioned media extracted from irradiated cells to examine the effects of media concentrations on MRC5 and QU-DB bystander responses. We hypothesized that if the negative feedback hypothesis is true, then the bystander response will increase owing to the reduction of bystander molecules (signals) in the conditioned medium. 

## Materials and Methods

### Cell culture

In this experimental study, two cell lines, a normal human lung fibroblast cell line (MRC5) and a human lung tumor cell line (QU-DB), were provided by Pasteur Institute, Tehran, Iran. MRC5 cells were grown in high glucose DMEM medium (Gibco, Germany) supplemented with 20% fetal bovine serum (Biosera, England) and 2 mM L-glutamine (Biosera, England). QU-DB cells were cultured in RPMI-1640 medium (Biosera, England) that contained 10% fetal bovine serum (Biosera, England), 100 U/ml penicillin (Biosera, England), and 100 μg/mL streptomycin (Biosera, England). The QU-DB cell line was cultured in 12 cm^2^ flasks, as well as 96- and 6-well plates and the MRC5 cell line was cultured in 12 cm^2^ flasks to prepare the experimental samples. The cells were kept in a CO_2_ incubator at 37˚C.

### Colony formation assay and irradiation

We prepared the directly irradiated groups by seeding QU-DB cells in 6-well plates at concentrations of 2×10^3^, 5×10^3^, 6×10^3^, 7×10^3^, and 8×10^3^ cells/well. After 24 hours, we irradiated the cells at 0 (control), 2, 4, 6, and 8 Gy gamma rays emitted from a Cobalt-60 teletherapy unit (Theratron, Phoenix model, average dose rate: 60-79 cGy/minutes) at room temperature. The field size was 15×15 cm^2^ with a source-to-surface distance of 70 cm. The target flasks were put in a water phantom (30×30×10 cm) used for dosimetry in order to include the scattering properties of the rays. Following irradiation, we returned the flasks to the incubator for 10 days (4).

Cells in the bystander groups were seeded in 12 cm^2^ flasks at a density 2×10^5^ cells/flask. At the same time, cells were also seeded in 12 cm^2^ flasks at a density of 2×10^5^ cells/flask as target cells. After 24 hours, we exposed the target cells to 0 (control), 2, 4, 6, and 8 Gy radiation. After irradiation, the flasks were placed in an incubator for 1 hour. Then, we extracted the conditioned media from the target flasks, filtered through 0.22 μm acetate cellulose filters and transferred to bystander cells. After a 24-hour incubation period, we removed the transferred media and the bystander cells were trypsinized. Then, the cells were seeded in 6-well plates at a density of 2×10^3^ cells/well and cultured for 10 days. Colonies were washed with PBS and fixed with pure methanol. After 24 hours they were stained with 10% Giemsa for 10 minutes. The number of colonies with more than 50 cells were counted and we calculated the percentage of the cells that survived (15).

### MTT assay and irradiation

In the MTT assay, the yellow tetrazolium MTT is reduced to purple formazan by metabolic activity in the mitochondrial cells. The resulting intracellular purple formation can be solubilized and measured by spectrophotometry. For MTT assay, we removed the culture media from the wells that contained the investigated cells. We added 10 μL of MTT solution (Sigma, St. Louis, MO) and 100 μL of fresh medium to separate wells, which were then allowed to incubate for 4 hours. After the incubation period, we replaced the culture media in the wells with 200 μL of Dimethylsulfoxide (DMSO, Sigma, USA) and plates were agitated for 10 minutes on a plate shaker to ensure adequate solubilization. Finally, the plates were read on a multi-well scanning spectrophotometer (Stat Fax 2100, USA) at a test wavelength of 545 nm (16). We measured and compared the percentage of viable cells to the control group.

Directly irradiated cell groups were seeded in 96- well plates at a density 4×10^3^ cells/well. After 24 hours, they were irradiated with 0 (control), 0.5, 2, 4, 6, and 8 Gy gamma rays. The MTT assay was performed, as described above, 5 days following irradiation. For bystander experiment, we cultured the target cells in 12 cm^2^ flasks at a density 2×10^5^ cells/ flask. One day later, they were irradiated. One hour after irradiation, the culture media from the irradiated flasks were extracted, passed through 0.2 μm filters (Orange Scientific, Belgium), and transferred to nonirradiated bystander cells which were seeded in 96 well plates. Then, the MTT assay was performed after 5 days for bystander cells in the same manner as the target irradiation cells.

### Medium dilution

We added fresh culture media to the conditioned media extracted from 4, 6, and 8 Gy irradiated cells in order to dilute them. 4 Gy irradiated media extracted from QU-DB target cells were diluted to 12 and 50% concentration and transferred to MRC5 bystander cells. 6 Gy irradiated media extracted from QU-DB target cells were diluted to 8, 34, and 67% concentration and transferred to autologous bystander cells. 8 Gy irradiated media extracted from QU-DB target cells were diluted to 6, 25, 50, and 70% and transferred to autologous bystander cells. Following the last medium transfer, we added cytochalasin B (Sigma-Aldrich, USA) to the QU-DB and MRC5 bystander cells (17) in order to perform the cytokinesis-blocked micronucleus assay. 

### Micronucleus assay

We performed the cytokinesis-block micronucleus assay to measure the numbers of cells that contained micronuclei. After the last medium transfer, the QU- DB bystander cells received 0.8 µg/ml of cytochalasin B. The MRC5 bystander cells received 2 µg/ml of cytochalasin B. QU-DB flasks that contained cytochalasin B were allowed to incubate for 45 hours, whereas MRC5 flasks were allowed to incubate for 24 hours (1.5 doubling time). Following incubation, the culture medium was removed, the cells in the flasks were washed with PBS, air-dried, and subsequently fixed. MRC5 cells were fixed once with absolute methanol and QU-DB cells with a combination of methanol:acetic acid (Merck, Germany) at a 3:1 ratio for three times. After drying, cells were stained with 10% giemsa for 5-6 minutes and viewed at ×400 magnification. For accuracy, one examiner scored the slides twice. Each time the number of cells that contained MNBN were counted (4). 

### Statistical analysis

All data obtained in this study had a normal distribution. Hence, we used one-way analysis of variance and Tukey’s multiple comparison tests at P<0.05 for statistical analyses. 

## Results

### Percentage of survival fraction and cell viability in directly irradiated and bystander cells at different radiation doses 

**Fig.1:**
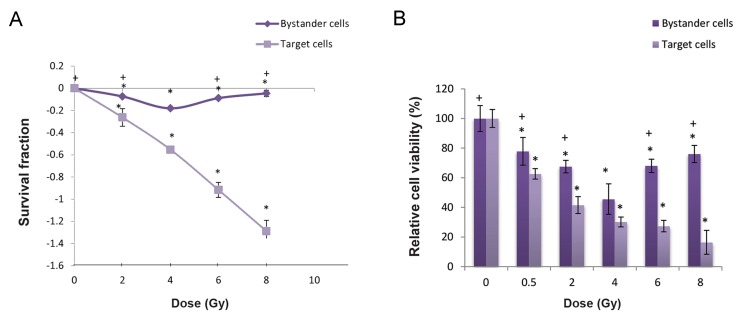
Survival fraction (SF) and relative cell viability of target and bystander QU-DB cells, exposed to different doses of gamma rays according to the colony assay (semi-logarithmic) and MTT assay. A. SF and B. Relative cell viability. *; Significant relationship between studied and control groups and +; Significant relationship between 4 Gy and other doses in bystander cells.

Figure 1 shows that the radiation response of QU-DB target and bystander cells according to the colony (semi-logarithmic scale) and MTT assays. The results have shown a consistent decrease of survival fraction (SF) with increasing dose. However, bystander cells did not follow the same response. SF initially decreased up to 4 Gy, then increased at 6 and 8 Gy. The percentage of cell viability in all groups compared to the controls statistically differed (P<0.001). A significant difference existed between 4 Gy and the other doses (P<0.001). 

**Fig.2:**
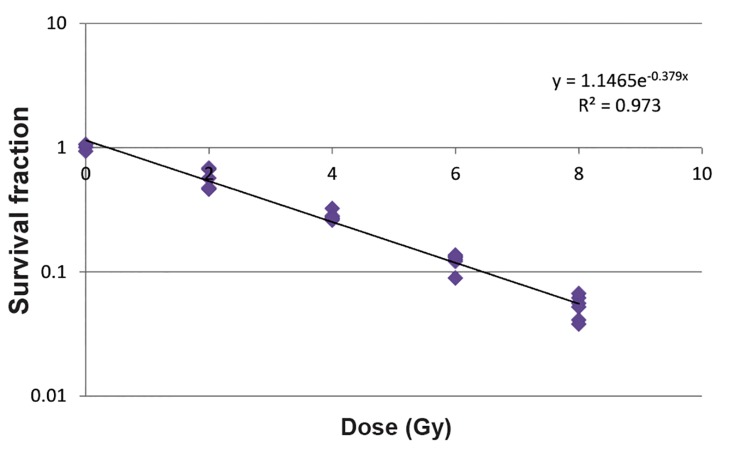
Logarithmic survival curve of QU-DB cell. Dose and survival fraction (SF) are plotted in linear and logarithmic scales, respectively. SF is an exponential function of dose. The dose to reduce survival to 37% of its value at any point on the final near exponential portion of the curve (D0) was 2.54 Gy.

Figure 2 shows a logarithmic survival curve for QU-DB target cells. In this curve, dose and SF were plotted in linear and logarithmic scales, respectively. SF was an exponential function of dose. The dose to reduce survival to 37% of its value at any point on the final near exponential portion of the curve (D0) was 2.54 Gy. 

### MNBN induction in QU-DB bystander cells 

**Fig.3:**
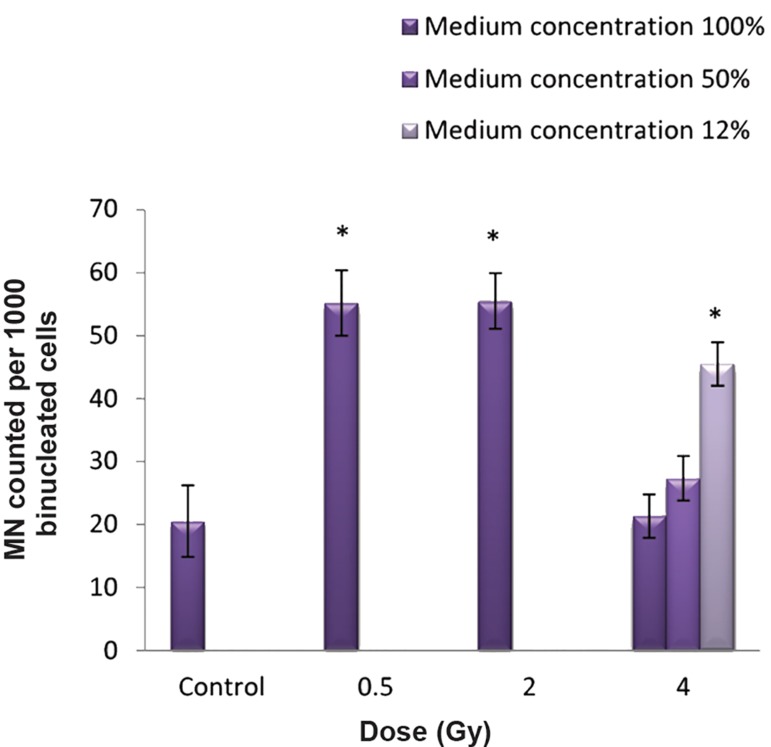
Number of micronucleated cells per 1000 binucleated cells (MNBN) of QU-DB cells that received diluted medium from QU- DB-irradiated cells at 6 and 8 Gy. Statistically significant differences existed in all subgroups compared with their control, except for 8 Gy.

Figure 3 shows the MNBN of different QU- DB bystander groups which received conditioned medium from autologous irradiated cells. Except for the 8 Gy group (P=0.511), all doses statistically differed compared to their control groups (P<0.05). There were statistically more MNBN cells counted in the 4 Gy group compared to the other doses (P<0.001) which supported the results of a previous study (13). Figure 3 shows the results of the medium dilution experiments as well. Conditioned media extracted from 6 and 8 Gy irradiated cells were mixed with fresh medium and transferred into autologous bystander flasks. Medium concentrations for 6 Gy were 8, 34, and 67%, whereas the medium concentrations for 8 Gy were 6, 25, 50, and 75%. Results indicated that diluted medium with 8% concentration of 6 Gy increased the MNBN frequency from 80.50 to 137 (P<0.0001). Medium dilution increased MNBN frequency in 75, 50, 25, and 6% diluted subgroups of 8 Gy from 78.50 to 145.66 (P<0.0001). Surprisingly, MNBN frequency of bystander cells which received 8 and 6% diluted medium (the least concentration) from 6 and 8 Gy irradiated cells respectively, reached the maximum level compared to MNBN frequency of the cells that received complete medium. A comparison of the subgroups indicated statistically significant differences in all subgroups compared with their controls, except for the 8 Gy complete medium subgroup. 

### MNBN induction in MRC5 bystander cells 

**Fig.4:**
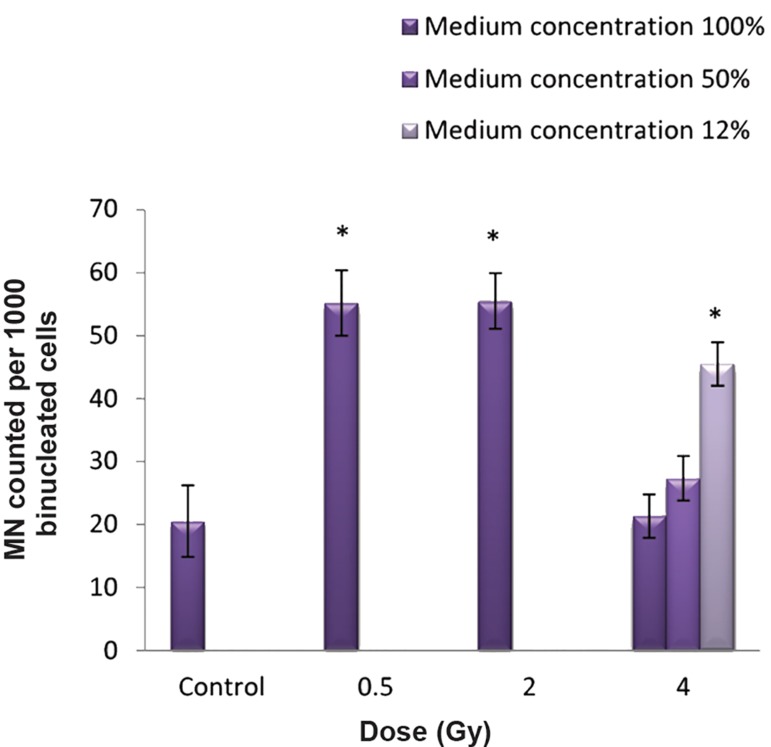
Number of micronucleated cells per 1000 binucleated cells (MNBN) when MRC5 cells received medium diluted from QU- DB-irradiated cells at 4 Gy. No statistically significant difference existed between the 100 and 50% (P=0.970) concentration groups. However, a statistically significant difference existed between the 100 and 12% concentration groups (P<0.001).

Figure 4 represents the number of MNBN cells in MRC5 bystander cells which received medium from QU-DB-irradiated cells. We observed statistically significant differences at 0.5 and 2 Gy compared with their controls, whereas no statistically significant differences existed between the 4 Gy and control groups. In the subgroups, we observed that the medium dilution did not affect the number of MNBN in the 50% diluted group at 4 Gy. Surprisingly, the MNBN frequency of bystander cells which received 12% diluted medium (the least concentration) reached the maximum level compared to the MNBN frequency of cells that received complete medium. Statistical analysis indicated no statistically significant difference between 100 and 50% (P=0.970) groups, whereas there was a statistically significant difference between the 100 and 12% concentration groups (P<0.001). 

**Fig.5:**
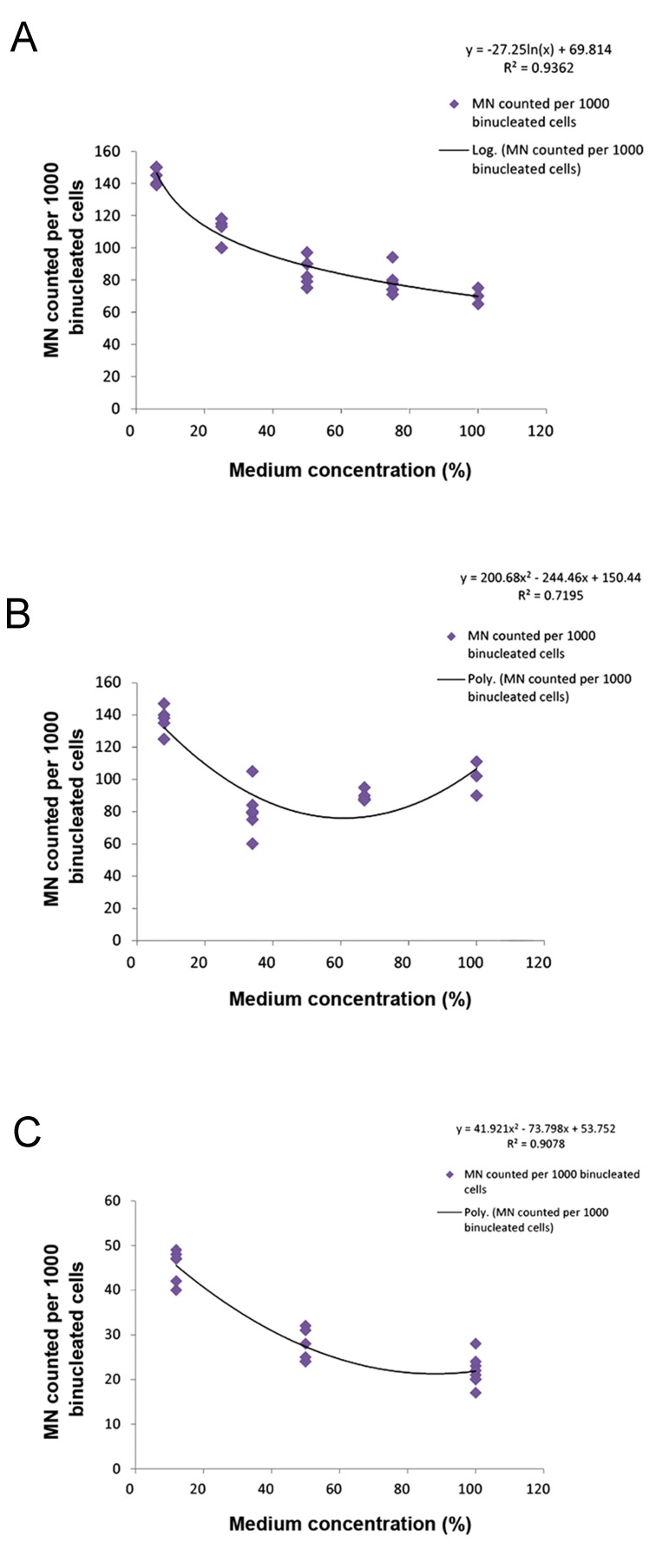
Regression test from different medium concentrations versus bystander response. A. Number of micronucleated cells per 1000 binucleated cells (MNBN) when QU-DB cells received medium diluted from QU-DB-irradiated cells at 8 Gy, B. MNBN when QU-DB cells received medium diluted from QU-DB- irradiated cells at 6 Gy, and C. MNBN when MRC5 cells received medium diluted from QU-DB-irradiated cells at 4 Gy.

In Figure 5, regression analysis showed a polynomial correlation between medium concentration and RIBE level for MRC5 cells and QU-DB cells at 4 and 6 Gy, respectively. However, the correlation between medium concentration and RIBE level for QU-DB cells at 8 Gy was logarithmic. 

## Discussion

The present study intended to clarify the uncertainties in RIBE observed at high radiation doses. In a prior study, the RIBE level (MNBN frequency) in QU-DB bystander cells increased at the dose ranges of 0.5 to 4 Gy, but decreased at higher doses (13). The number of MNBN cells in MRC5 bystander cells which received medium from QU-DB-irradiated cells increased in the dose ranges of 0.5 to 2 Gy, but decreased at 4 Gy. We expected a higher RIBE level at the high dose. The data obtained in the previous study appeared to be insufficient to explain the observations. 

In the present study we evaluated RIBE levels with other endpoints: mitotic death/SF and proliferation/cell viability by the colony assay and MTT test. The results of this study confirmed the previous study’s results (13). When conditioned medium from 4 Gy irradiated QU-DB cells were transferred to MRC5 cells, the bystander effect disappeared and the number of micronucleated cells decreased to control levels. In the case of QU-DB cells, when the dose increased to 4 Gy, the SF of QU-DB bystander cells decreased. However, we observed an SF increase above 4 Gy. Therefore, 4 Gy was a critical dose which induced maximum RIBE in QU-DB cells. This observation indicated that not only MNBN frequency was an end-point, but also RIBE as a whole decreased at 6 and 8 Gy. In the previous study, the results of the micronucleus assay relied on cell replication status. We investigated the Nuclear Division Cytotoxicity Index (NDCI) of all cell groups in order to verify whether MNBN reduction was the result of a cell replication delay or not. The results indicated that MNBN reduction was not due to delays in replication (13). 

Other researchers observed decreased RIBE at high doses. Boyd et al. (18) reported that at doses below 2 Gy, the bystander effect induced by alpha and auger electrons was proportional to the dose. However, above 2 Gy, they observed a decrease in bystander effect. Gow et al. (2) used 20 MeV electrons and Cobalt-60 gamma rays at doses of 0.5, 5, and 10 Gy to investigate bystander response in HPV-G cells. In their study, media from 0.5 and 5 Gy irradiated cells transferred to bystander cells caused a decrease in the numbers of bystander cells that survived. However, when the dose increased to 10 Gy, RIBE was abrogated in both electron particles and gamma ray cases. The authors proposed that a negative feedback mechanism caused an increased survival at 10 Gy. They explained that the fraction of surviving cells decreased in a dose-dependent manner until a saturation occurred, with subsequent recovery and repair at high doses of irradiation. This precisely correlated with the behavior of transforming growth factor (TGF)-beta. Small amounts of active TGF-beta produced at low doses led to a relatively small amount of active TGF-beta that bound to each recipient. They supposed as the dose increases, more TGF-beta are produced and conjugate to bystander cells, until all receptors on bystander cells’ membrane are occupied. In this situation excess TGF-beta molecules, produced by high doses, cannot bound to bystander cells. Hence, RIBE saturates or a negative feedback occurs. 

In the current study, we examined this negative feedback hypothesis. We diluted the conditioned media extracted from 6 Gy irradiated QU-DB cells with fresh medium, so that their concentration decreased to 8, 34, and 67% of the complete conditioned medium. Similar procedure was performed for the conditioned media extracted from 8 Gy irradiated QU-DB cells to provide 6, 25, 50, and 75% diluted media. These media were subsequently transferred to autologous bystander cells. Surprisingly, in contrast to the low level of MNBN frequency in QU-DB bystander cells at 6 and 8 Gy, MNBN values in bystander cells of diluted medium (at least concentration, 8% diluted medium from 6 Gy and 6% diluted medium from 8 Gy) reached to a maximum level. The same experiment for MRC5 bystander cells revealed, when medium extracted from QU-DB target cells was diluted to 12%, RIBE revived and MNBN frequency increased. These results confirmed the existence of a negative feedback at high doses (4 Gy for MRC5 cells and 6 and 8 Gy for QU-DB cells), which could be abolished with condition medium dilution. 

Mackonis et al. (14) investigated cell SF in the shielded and unshielded areas of fields of modulated beams and com¬pared this with cell SF in uniform control fields. When unshielded areas were irradiated, the survival of shielded areas increased more than the equal control fields. They suggested that the process of cell death in the unshielded areas was related to stimulation of a repair mechanism in the viable cells in the shielded areas. However, Blyth and Sykes (9) suggested that target cell death at a high dose caused a reduction in the number of bystander signals, and consequently at the RIBE level. Their hypotheses could not explain the current study observations. 

Since, if signals were too low to induce RIBE, we could not revive it with medium dilution. 

In our previous study, the MNBN frequency of QU-DB bystander cells which received diluted medium from 4 Gy QU-DB irradiated cells was not statistically different from MNBN frequency of cells that received complete medium from 2 Gy QU-DB irradiated cells (19). This investigation demonstrated that the quantity of bystander signals in 2 Gy irradiated cells was the same as the amount of signals which existed in the 50% diluted medium concentration from 4 Gy irradiated cells. We suggested that a direct correlation existed between dose and the quantity of bystander signals generated by target cells (19). In contrast, Baskar et al. (12) reported that medium dilution decreased RIBE response of GM637H cells, whereas the RIBE cell response was independent of dose. We hypothesized a dose-dependent increase in the amount of signals produced by QU-DB target cells (19). We also suggested that the type of signals may be different at high dose. Therefore, it is likely that different molecular pathways were activated at different doses and acted as intracellular or intercellular signal carriers. Hence, this led to the induction of different amounts of RIBE in bystander cells. As previously mentioned, we have observed decreased numbers of micronucleated cells in MRC5 bystander cells at the 4 Gy dose to QU-DB cells. If the same decreased RIBE occurs in stereotactic radiotherapy, brachytherapy, and hypofractionated protocols which apply high doses per fraction (20), normal tissues can be protected, in contrast to our expectation. It is likely that RIBE due to the high dose applied in these modalities is different and influences their radiobiological outcomes. Consequently, more studies should discuss this issue. 

## Conclusion

Our results showed that RIBE levels decreased at doses above 4 Gy; however, RIBE levels increased when diluted conditioned medium was transferred to bystander cells. The results confirmed that a negative feedback mechanism was responsible for decreased RIBE response at high doses. Decreased RIBE at high doses might be used to predict normal tissue damage as a result of RIBE in radiotherapy modalities such as radiosurgery, brachytherapy and grid therapy that use high dose fractionation. Based on our results and those from other studies, we predict that the impact of high dose radiation on RIBE depends on cell type and total dose applied. Therefore, we have proposed that a variety of normal/tumor cells and molecular pathways should be considered for future research. It is necessary to perform research on animal models which are preferred to cell cultures that are far from real radiotherapy conditions. 
